# PhiC31 integrase induces a DNA damage response and chromosomal rearrangements in human adult fibroblasts

**DOI:** 10.1186/1472-6750-9-31

**Published:** 2009-04-02

**Authors:** Jian Liu, Tina Skjørringe, Torben Gjetting, Thomas G Jensen

**Affiliations:** 1 The Kennedy Center, Gl. Landevej 7, 2600 Glostrup, Denmark; 2 Department of Laboratory Medicine, Clinical Research Center, Malmö University Hospital, 20502 Malmö, Sweden; 3 Department of Radiation Biology 6321, Finsen Centre, Rigshospitalet, 2100 Copenhagen Ø, Denmark; 4 Institute of Human Genetics, University of Aarhus, Aarhus, Denmark

## Abstract

**Background:**

PhiC31 integrase facilitates efficient integration of transgenes into human and mouse genomes and is considered for clinical gene therapy. However recent studies have shown that the enzyme can induce various chromosomal abnormalities in primary human embryonic cells and mammalian cell lines. The mechanisms involved are unknown, but it has been proposed that PhiC31 attachment sites in the host genome recombine leading to chromosomal translocations.

**Results:**

We have studied possible effects of the PhiC31 integrase expression in human adult fibroblasts by karyotyping. All control cells were cytogenetically normal, whereas cells expressing PhiC31 integrase show chromosomal abnormalities confirming our previous results using primary embryonic fibroblasts. In order to study the early mechanisms involved we measured H2AX phosphorylation – a primary event in the response to DNA double-strand-breaks. Transient transfection with PhiC31 integrase encoding plasmids lead to an elevated number of cells positive for H2AX phosphorylation detected by immunofluorescence. Western blot analysis confirmed the upregulated H2AX phosphorylation, whereas markers for apoptosis as well as p53 and p21 were not induced. Cells transfected with plasmids encoding the Sleeping Beauty transposase remained cytogenetically normal, and in these cells less upregulation of H2AX phosphorylation could be detected.

**Conclusion:**

In primary human fibroblasts expression of PhiC31 integrase leads to a DNA damage response and chromosomal aberrations.

## Background

PhiC31 integrase, originally isolated from *Streptomyces lividans*, is widely used for non-viral gene delivery and vector integration [1]. PhiC31 integrase mediated integration leads to prolonged gene expression and has been used for correction of disease models [2,3]. However, it has been reported that PhiC31 integrase can lead to genomic deletions, chromosomal rearrangements and chromosomal instability [4-8]. In mouse cells it was recently shown that the integrase does lead to imprecise deletion of self excision cassettes [9]. In cell lines, PhiC31-mediated integration of plasmid DNA may be accompanied by chromosomal rearrangements in the mammalian host genome with a frequency up to 15% [10]. The mechanisms involved are unknown, but it has been speculated that cryptic PhiC31 attachment sites recombine leading to chromosomal translocations [10]. In contrast to this, the system for transposon-directed genomic integration facilitated by the transposase Sleeping Beauty apparently does not cause chromosomal aberrations [8]. In order to assess the mechanistic and potential harmful effects of cellular expression of PhiC31 integrase and Sleeping Beauty we have studied the immediate DNA damage responses and the long-term effect of induction of genomic rearrangements.

## Results and discussion

### Generation of primary human fibroblasts expressing PhiC31 integrase

In gene transfer applications, the PhiC31 integrase mediates the integration of plasmids bearing an *attB *site into sequences with partial sequence identity to *attP *(pseudo-*attP *sites). Primary adult human fibroblasts were co-transfected with the plasmids pBabepuro or pBabepuroatt, containing the 285-base pair *attB *sequence, in combination with the plasmid pCMV-Int encoding the PhiC31 integrase. Transfections were performed in combination with a 3:1 molar excess of pCMV-Int compared to the pBabepuro/pBabepuroatt plasmids in order to increase the likelihood that pCMV-Int plasmids were integrated in the genome. After puromycin selection, DNA and RNA were isolated for PCR analysis using primers specific for the integrase gene. In this analysis clear signals were detected from puromycin selected cells transfected the pCMV-Int plasmid showing that the integrase is exclusively present and expressed in cells transfected with pCMV-Int (data not shown).

### Cytogenetic analysis

The karyotypes of puromycin selected cells are shown in Table 1 (left column, transfection experiment 1). Cells transfected with pBabepuro or pBabepuroatt alone had normal karyotypes. In contrast, abnormal karyotypes including aneuploidy and deletions were found in cells co-transfected with the pCMV-Int plasmids confirming our previous results with primary embryonic cells [6]. The data in Table 1, right column show evaluation of effect of expression of the Sleeping Beauty transposase, as will be described below.

**Table 1:** Karyotypes of primary human fibroblasts

	**Cytogenetic analysis**	
	
**Transfected constructs**	**Transfection experiment 1**	**Transfection experiment 2**
pBabepuro	46, XY (n = 10)	46, XY (n = 15)
pBabepuroatt	46, XY (n = 10)	46, XY (n = 18)
pBabepuro + pCMV-Int	46, XY (n = 6)	
	Extra chr. 10 (n = 1)	
	Del(1)(p11) (n = 1)	
	Add 17q (n = 1)	
	Chr. 2 loss (n = 1)	
pBabepuroatt + pCMV-Int	46, XY (n = 6)	
	Extra chr. (n = 1)	
	Ring Chr. No. 7 (n = 2)	
	Del(13)(q21.1) (n = 1)	
pBabepuro + pCMV-SB		46, XY (n = 17)
pBabepuro + pCMV-mSB		46, XY (n = 2)
pBabepuro-SB*		46, XY (n = 16)
pBabepuro-mSB*		46, XY (n = 15)

Number of cells analysed (n) are indicated in parentheses. *The expression cassettes for SB or mutant SB (mSB) were cloned into the pBabepuro plasmid. Metaphase chromosomes were analyzed by standard Q-banding techniques. The description of chromosome aberrations was based on the recommendations of the International System for Human Cytogenetic Nomenclature (ISCN 1995).

### Analysis of histone H2AX phosphorylation

**Figure 1:**
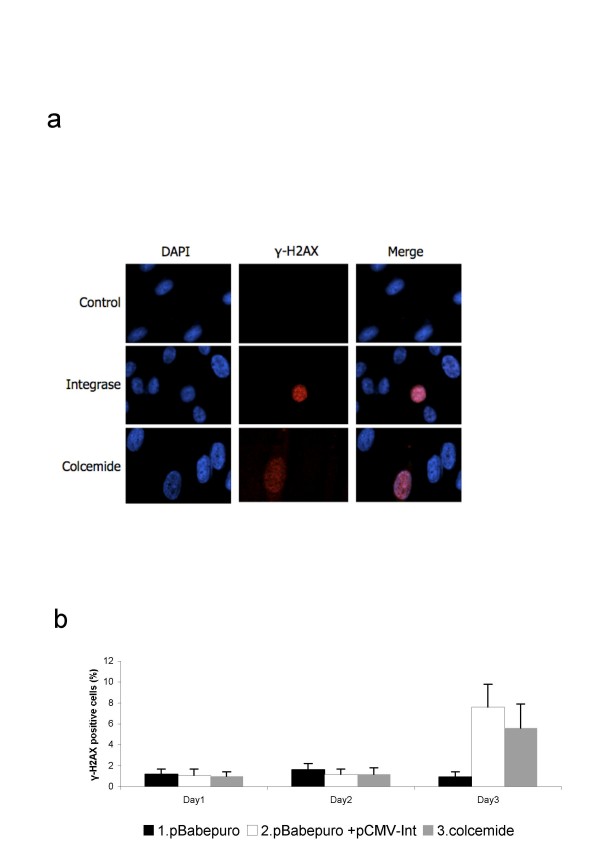
**Analysis of H2AX phosphorylation**. a) Immunofluorescence analysis of NHDF cells transfected with pBabepuro alone (control) or in combination with pCMV-Int (integrase). Blue color is DAPI-staining, visualizing nuclear DNA. Red color is staining of phosphorylated H2AX using anti-γ-H2AX antibodies. b) Quantitation of the percentage of cells with upregulated H2AX phosphorylation. Each experiment was repeated 3 times, and 100 cells were counted in each sample. Error bars indicate standard error of the mean. Only in day 3 there is a significant difference between the three treatments (p < 0.05).

**Figure 2:**
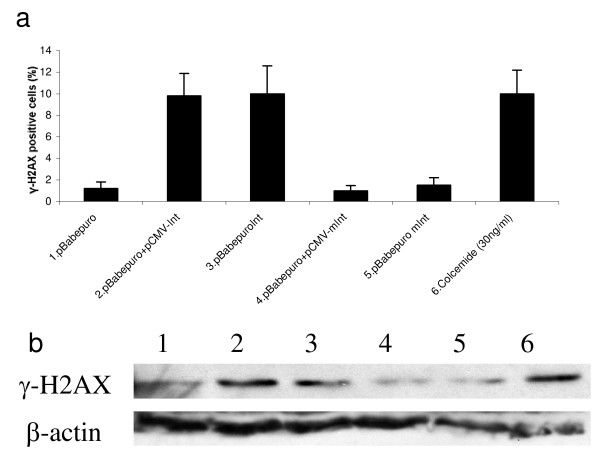
**H2AX phosphorylation 3 days after transfection of NHDF cells**. a) Immunofluorescence analysis. b) Western blotting analysis. Lanes: 1) pBabepuro 2) pBabepuro+pCMV-Int 3) pBabepuroInt 4) pBabepuro+ pCMV-mInt 5) pBabepuromInt 6) Colcemide control experiment causing DNA damage.

The histone H2AX is a key protein of the cellular response to DNA damage and becomes rapidly phosphorylated in response to DNA damage [11,12]. Phosphorylated H2AX can be detected using immunofluorescence leading to clear and distinct nuclear staining (Fig. 1a). Normal Human Dermal Fibroblasts (NHDF) treated with colcemide, serving as a positive control [13], or transfected with pBabepuro or pBabepuro+pCMV-Int were analysed for H2AX phosphorylation at various times after transfection. As seen in Fig. 1b, cells transfected with pBabepuro+pCMV-Int show an approx. 4 fold increase in the number of nuclei positive for H2AX phosphorylation three days after the treatment. This is comparable to the number of stained nuclei resulting from colcemide treatment. Cells only transfected with the control plasmid pBabepuro showed no increase in the fraction of cells positive for H2AX phosphorylation. The increase was detected in cells transfected with both plasmids pCMV-Int and pBabepuroInt, but not after transfection with mutant PhiC31 genes (Fig 2a). The upregulated H2AX phosphorylation was confirmed by Western blotting analysis (Figure 2b). Here, in cells transfected with pBabepuro in combination with pCMV-Int (lane 2) or one plasmid containing gene expression cassettes for both puromycin-resistance and integrase (lane 3) clear bands representing phoshorylated H2AX is detected. Band intensity is comparable to cells treated with colcemide (lane 6), whereas cells transfected with the control plasmids pBabepuro alone (lane 1) or plasmids expressing an inactive mutant variant integrase, pBabepuro+pCMV-mInt (lane 4) and pBabepuromInt (lane 5) show only weak bands of gamma-H2AX.

**Figure 3:**
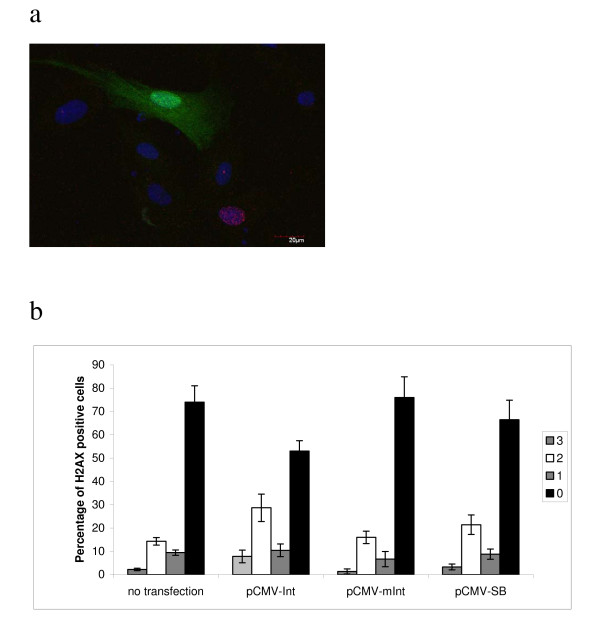
**Analysis of H2AX phosphorylation using co-transfection of plasmids encoding EGFP and PhiC31 integrase or Sleeping Beauty (SB) transposase**. a) Cells co-transfected with plasmids encoding EGFP (green) and integrase were immunostained using anti-γ-H2AX antibodies (red). b) H2AX immunostaining was quantified by ranking H2AX positive cells from 1 to 3, 3 being the very intensive nuclear staining and 1 being the vague but yet defined nuclear staining. Staining intensities were quantified in GFP positive cells.

Using immunofluorescence the degree of H2AX phosporylation was scored by an arbitrary range ranking of staining intensities. Score 3 was defined as the very intensive nuclear staining, score 2 intermediate, and score 1 being the faint but yet defined nuclear staining (Fig. 3). NHDF cells transfected with pCMV-Int had significantly more cells positive for H2AX phosphorylation scored as 3 and 2 compared to non-transfected cells (*P *= 0.0015, Fisher's exact test). Immunofluorescence analysis of transfected cells did not reveal upregulation of p53 and p21, and early markers for apoptosis were not significantly upregulated (data not shown). Collectively, these results suggest that active PhiC31 integrase leads to a DNA damage response in transfected NHDF cells.

### Analysis of possible effects of expression of SB transposase

Plasmids encoding wildtype and mutant SB transposase were co-transfected with pBabepuro and the cells subsequently selected in puromycin. After drug selection the chromosomes were analysed using G-banding. As seen in Table 1 (right column) all puromycin selected cells had normal chromosomes. After transient transfection the SB transposase resulted in lower levels of H2AX phosphorylation compared to cells transfected with the PhiC31 integrase plasmids measured by immunofluorescence (Fig. 3).

Altogether we have shown that the PhiC31 integrase induces chromosome rearrangements in primary human adult cells confirming our previous results using primary human embryonic fibroblasts [6]. The observed effects take place even in the absence of donor plasmids containing attB sequences indicating that the effects are caused by the integrase enzyme directly. Furthermore we show that the PhiC31 integrase, in contrast to the SB transposase, leads to significant up-regulation of H2AX phosphorylation. Interestingly, immunofluorescence staining reveal numerous bright spots in the nucleus in a pattern similar to that observed when cells are treated with colcemide, which causes DNA damage in multiple loci. H2AX phosphorylation leads to recruitment of repair factors to damaged DNA [14] and efficient homologous recombinational repair of chromosomal double-strand breaks [15] independent of p53 [16].

H2AX phosphorylation was observed already 3 days after transfection, whereas the abnormal karyotypes were measured after drug selection (15–17 days after transfection). The inducible PhiC31 integrase system described recently may be used to establish whether the two events, DNA damage and chromosome rearrangements, are causally linked [17]. Moreover, the dosage dependent effects of integrase are still unknown and need to be further clarified. Our findings highlight the importance of screening for chromosomal rearrangements in mammalian cells and investigation of the genetic and phenotypic consequences.

## Conclusion

In primary human adult fibroblasts PhiC31 integrase leads to a DNA damage response and chromosomal aberrations. Our findings have important implications for gene therapy approaches based on PhiC31 emphasising that safety issues for gene therapy approaches need to be carefully addressed.

## Methods

### Plasmid constructs and cell lines

The plasmid pBabepuro containing a puromycin selection marker has been described before [18]. The plasmid pBabepuroatt contains the specific 285 bp bacterial attachment site for PhiC31 integrase [6]. Plasmid pCMV-Int harbouring the PhiC31 integrase gene driven from a CMV promoter was a generous gift from Dr. Calos, Stanford University, CA, USA. The coding sequence of PhiC31 integrase was excised from pCMV-Int by *Pst*I and *Bam*HI digestion and ligated into pBabepuro to create the plasmid pBabepuroInt. The plasmids pCMV-mInt and pBabepuromInt were developed by deleting a 537 bp in-frame fragment in the integrase gene using the restriction enzyme *Pml*I (New England Biolabs, USA). The deletion reduces the polypeptide length from 613 amino acid residues (68 kDa) to 434 amino acid residues (48 kDa) and encompasses the entire DNA binding region of the protein rendering it inactive. The plasmid pCMV-SB, expressing the SB transposase (SB10) from the CMV immediate-early promoter, and pCMV-mSB expressing an inactive variant of the SB transposase have been described previously [19]. The pEGFP-N1 plasmid contains a variant of GFP, EGFP, driven by a CMV immediate-early promotor (CLONTECH Laboratories, Inc., USA).

NHDF were purchased from Cambrex Corporation. The cells were cultivated in Dulbecco' s modified Eagle medium (Invitrogen) supplemented with 10% fetal bovine serum (GIBCO) and 1% Penicillin-streptomycin (GIBCO).

### Transfections

Cells that had reached 50–80% confluency in 25 cm^2 ^flask were transfected with plasmids pBabepuroInt and pBabepuromInt alone, or pBabepuro and pBabepuroatt containing integrase and/or the recognition site by using FuGene 6 transfection reagent (Roche) at a ratio of 3 μl of FuGene 6 transfection reagent per μg of DNA. For stable transfection cells were subjected to puromycin selection (5 μg/ml) 24 hrs after transfection.

### PCR and RT-PCR analysis of integrase

Genomic DNAs were extracted from transfected cells using a Genomic DNA purification kit (Gentra Systems). RNA was extracted using RNeasy Mini Kit (Qiagen). DNA was analyzed by polymerase chain reaction using primers for the integrase gene, forward primer 5'-ACTCGACCACTTCCCTTACC-3' and reverse primer 5'-ACCACGCCTGAAGCTCATAC-3'. RNA was isolated by an RNeasy kit (Qiagen) and reverse transcribed to cDNA using first strand cDNA synthesis kit for RT-PCR (AMV) (Roche). Subsequently, the cDNA was subjected to PCR analysis using the primers mentioned above.

### Cytogenetic analysis

Metaphase chromosomes were analyzed by standard Q-banding techniques 15–17 days after transfection. The description of chromosome aberrations was based on the recommendations of the International System for Human Cytogenetic Nomenclature (ISCN 1995) [20].

### Immunofluorescence analysis

Cells were grown on 12 mm glass coverslips in 6 well plates. After transient transfection the coverslips were briefly washed in cold PBS, fixed for 10 min at room temperature with ice cold ethanol/methanol (1:1, v:v). After drying, the coverslips were stained using anti-γ-H2AX antibodies (Abcam, Cambridge, MA, USA) and Texas red-anti-rabbit secondary antibodies (Abcam) with 10 min PBS washing steps and finally evaluated by epifluorescence microscopy.

Alternatively, when detection of fluorescent proteins in transfected cells was needed, a paraformaldehyde fixation method was used. Briefly, cells for ranking of γ-H2AX expression were grown in 4-chamber Labtech glass slides (NUNC, Denmark). Three days after transfection with pIntegrase+pEGFP-N1+pBabepuroattB, pIntegrasemut+pEGFP-N1+pBabepuroattB, and pCMV-SB+pEGFP-N1+pBabepuroattB, respectively (2:2:1), the cells were briefly washed in PBS and fixed for 15 minutes in cold 2% paraformaldehyde. Cells were permeabilised using 0.2% saponin in the blocking buffer (PBS, 1% BSA). Staining was done using anti-γ-H2AX antibodies (Abcam) 1:1000 and secondary anti-rabbit Alexafluor 594 (Invitrogen Inc.) diluted 1:1000. Slides were mounted in Vectashield and counterstained with 4', 6-diamidino-2-phenylindole (DAPI; Vector Laboratories, Burlingame, CA) and analysed using a Fluoview FV1000 confocal microscope (Olympus). Empirical ranking of staining intensities were scored as 1 to 3, 3 being the very intensive nuclear staining, 1 being the vague but yet defined nuclear staining. Cells were ranked in 48 to 68 independent windows. In total 115 (pCMV-Int), 182 (pCMV-SB) 75 (pCMV-mInt) and 1174 (no transfection) cells were monitored and scored.

p53 and p21 were measured using immunofluorescence (Abcam, Cambridge, UK) and apoptosis detected using an In Situ Cell Death Detection Kit (Roche).

### Western blot analysis

At day 3 after transfection, cells were washed twice in PBS and lysed using Laemmli sample buffer as described by Harlow and Lane, Antibodies, 2^nd ^ed. Briefly, aliquots were boiled at 95°C for 10 min and loaded onto 12% acrylamide gels. After eletrophoresis, proteins were transferred to HybondECL membranes (GE Healthcare) by semidry transfer. The membrane was blocked with PBST (PBS containing 0.1% Tween 20) containing 5% nonfat milk for 60 minutes before incubation with 50 ng/mL anti-γ-H2AX antibody (Abcam, Cambridge, UK) overnight at 4°C in a humidified chamber. The membrane was washed in PBST and then incubated with horseradish peroxidase-conjugated anti-rabbit antibody (1/1000) dilution for 1 hr at the room temperature and visualized by chemiluminescence using ECL Plus Western Blotting Detection System (GE Healthcare).

## Authors' contributions

JL performed the plasmid constructions, most transfections, western blotting and immunofluorescence. TS performed the experiments with co-transfection with EGFP including analysis of H2AX phosphorylation in these experiments. TG supervised JL and TS and performed the statistical analysis. JL drafted the manuscript along with TS and TGJ. TGJ participated in experimental designs and finalised the manuscript. All authors read and approved the final manuscript.
